# Fibroid explants reveal a higher sensitivity against MDM2-inhibitor nutlin-3 than matching myometrium

**DOI:** 10.1186/1472-6874-12-2

**Published:** 2012-01-10

**Authors:** Dominique N Markowski, Burkhard M Helmke, Arlo Radtke, Jennifer Froeb, Gazanfer Belge, Sabine Bartnitzke, Werner Wosniok, Iris Czybulka-Jachertz, Ulrich Deichert, Jörn Bullerdiek

**Affiliations:** 1 Center of Human Genetics, University of Bremen, Leobener Strasse ZHG, D-28359 Bremen, Germany; 2 Institute of Pathology, University of Heidelberg, Im Neuenheimer Feld 220/221, D-69120 Heidelberg, Germany, current address of B.M.H.: Institute of Pathology, Elbe Kliniken, Klinikum Stade, Bremervörder Str. 111, D- 21682 Stade, Germany; 3 Institute of Statistics, University of Bremen, Am Fallturm 1, 28359 Bremen, Germany; 4 Clinic of Gynecology and Obstetrics, Elbe Kliniken, Klinikum Stade, Bremervörder Str. 111, 21682 Stade, Germany; 5 Clinic of Gynecology and Obstetrics, Krankenhaus Cuxhaven, Altenwalder Chaussee 10, D-27474 Cuxhaven, Germany; 6 Small Animal Clinic, University of Veterinary Medicine, Bünteweg 9, D-30559 Hannover, Germany

**Keywords:** fibroids, p14^Arf^, senescence, apoptosis, MDM2 antagonists

## Abstract

**Background:**

Spontaneous cessation of growth is a frequent finding in uterine fibroids. Increasing evidence suggests an important role of cellular senescence in this growth control. Deciphering the underlying mechanisms of growth control that can be expected not only to shed light on the biology of the tumors but also to identify novel therapeutic targets.

**Methods:**

We have analyzed uterine leiomyomas and matching normal tissue for the expression of p14^Arf ^and used explants to see if reducing the MDM2 activity using the small-molecule inhibitor nutlin-3 can induce p53 and activate genes involved in senescence and/or apoptosis. For these studies quantitative real-time RT-PCR, Western blots, and immunohistochemistry were used. Statistical analyses were performed using the student's *t *test.

**Results:**

An in depth analysis of 52 fibroids along with matching myometrium from 31 patients revealed in almost all cases a higher expression of p14^Arf ^in the tumors than in the matching normal tissue. In tissue explants, treatment with the MDM2 inhibitor nutlin-3 induced apoptosis as well as senescence as revealed by a dose-dependent increase of the expression of *BAX *as well as of *p21*, respectively. Simultaneously, the expression of the proliferation marker Ki-67 drastically decreased. Western-blot analysis identified an increase of the p53 level as the most likely reason for the increased activity of its downstream markers *BAX *and *p21*. Because as a rule fibroids express much higher levels of p14^Arf^, a major negative regulator of MDM2, than matching myometrium it was then analyzed if fibroids are more sensitive against nutlin-3 treatment than matching myometrium. We were able to show that in most fibroids analyzed a higher sensibility than that of matching myometrium was noted with a corresponding increase of the p53 immunopositivity of the fibroid samples compared to those from myometrium.

**Conclusions:**

The results show that uterine fibroids represent a cell population of advanced cellular age compared to matching myometrium. Moreover, the data point to members of the p53-network as to potential novel therapeutic targets for the treatment of uterine fibroids.

## Background

Uterine fibroids are by far the most common gynaecological tumors at all. In premenopausal women incidences of 60-70% have been reported [1,2] Although only a minority of the leiomyomas become symptomatic the presence of symptomatic leiomyomas is still the leading cause for hysterectomy worldwide. Despite their high prevalence the treatment options besides surgical removal by hysterectomy or tumor enucleation are still limited. Treatment by GnRH agonists as well as antagonists can induce shrinkage of fibroids but re-growth of the tumors usually occurs after termination of the therapy [3,4]. Thus, intervention at the hormonal level is as a rule only recommended to reduce tumor size pre-operatively [5]. Another alternative represents embolization of the fibroids but the recurrence of myoma-related symptoms is not a rare finding after that treatment as well [6]. Thus, therapies aimed at permanent shrinkage of the fibroids still remain a challenge. Recently, we have presented evidence that in leiomyoma development the overexpression of *p14^Arf ^*drives a negative feedback-loop between p53 and MDM2 that governs the fate of the individual fibroid. Compared to matching myometrial tissue the myomas display a significantly higher expression of one of the genes of the senescence associated *Ink4a/Arf *locus i.e. *p14^Arf ^*[7]. It is not clear yet if this elevated expression solely results from an enhanced proliferative activity of the fibroid compared to its tissue of origin or if the same oncogenic stimuli triggering the leiomyoma growth do simultaneously stimulate *p14^Arf ^*as an oncogene-induced senescence-like mechanism. However, whatever is the cause of the p*14^Arf ^*overexpression it activates a p53-MDM2 negative feedback-loop [8] that may govern a delicate balance of the fibroids between proliferative activity and senescence [7]. This makes antagonizing MDM2 an interesting approach towards the growth control of fibroids. Accordingly, we have used nutlin-3, a known MDM2 inhibitor, to antagonize its activity in cell cultures from fibroids. Interestingly, we were able to show that antagonizing MDM2 induces the activity of genes associated with senescence (*p21*) as well as those with apoptosis (*BAX) *in leiomyoma cells *in vitro *[9]. Also, the MDM2 inhibitor drastically reduced cell proliferation as indicated by a decreasing level of *Ki-67 *expression. Nevertheless, the question arises if fibroids display a higher sensitivity than matching myometrium as can be suggested from their higher expression of *p14^Arf ^*[7] or the spontaneous senescence observed in fibroids [10]. Herein, we have used tissue explants taken from leiomyomas and matching myometrium to analyze the effects of an MDM2 antagonist and possible different sensitivities of the fibroids and their matching tissue of origin.

## Methods

### Tissue Samples

Altogether, tissue samples of 36 patients have been investigated in this study (Table 1). The study was approved by the local ethics committee (Ethikkomission bei der Ärztekammer Bremen) and prior to surgery, informed written consent was obtained from all patients. For gene expression studies samples of 52 UL from 31 patients along with matching myometrium were taken during surgery, immediately frozen in liquid nitrogen, and stored at -80°C for RNA isolation and qRT-PCR analyses of p14^Arf^. For MDM2 inhibition six tissue samples of UL from four patients as well as matching myometrium were taken during surgery and immediately transferred into sterile Hank's solution.

**Table 1:** Age of the patients, tumor size and karyotype of the leiomyomas investigated.

**case no**.	Age	tumor-size [cm]	karyotype
0501-1	48	8.0	46, XX [36]

0503-1	40	4.0	46, XX, inv(5)(q15q31~33), t(12;14)(q15;q24)[13]

0504-1	43	2.0	46, XX [11]

0515-1	46	3.0	n.d.

0529-1	44	7.0	46, XX [12]
0529-2	44	5.0	46, XX [14]
0533-1	41	6.0	46, XX, r(1), t(1;12;14)(p36.3;q14;q24)[19]

0535-1	43	5.0	47, XX, +10 [2]/46, XX [10]
0535-2	43	4.0	46, XX, t(8;11)(p23;q13.1)[6]/47, XX, +12 [2]/46, XX [15]
0535-3	43	3.0	46, XX [7]
0535-4	43	2.0	46, XX [15]
0535-5	43	2.0	46, XX, del(7)(q11.2?)[2]/46, XX [12]

0538-4	36	3.0	46, XX [6]
0540-1	49	4.0	46, XX [10]
0540-2	49	N/A	46, XX [4]
0541-1	37	7.0	46, XX, t(12;14)(q15;q24)[5]/46, XX [9]
0545-1	47	5.0	46, XX, t(12;14)(q15;q24)[9]/46, XX [3]

0549-2	49	3.5	46, XX [10]
0549-3	49	4.0	46, XX [10]
0549-4	49	6.0	48, XX, +der(6), -8, +11, +mar [11]

0556-1	42	5.0	46, XX, t(3;5;12)(q25;p14;q15)[11]/45, XX, t(3;5;12)(q25;p14;q15), -22 [10]

0557-1	38	1.0	46, XX [10]

0561-1	44	15.0	n.d.

0579-1	49	1.5	46, XX, t(12;15;14)(q15;q26;q24)[20]

0583-1	40	5.5	46, XX [16]

0596-1	49	8.5	46, XX, ins(2;12)(q34 or q35;q24.3 or q24.1q13), inv(4)(q27q31.3)[22]

0628-1	57	4.0	46, XX, t(2;4)(q33;q25)[14]
0628-2	57	1.5	46, XX, ?ins(12;14)(q15;q31q24)[5]/46, XX [14]

0632-1	47	4.0	46, XX, t(12;14)(q15;q24)[12]/46, XX, del(4)(q31orq32), der(10), ?t(10;14)(q24;q32), t(12;14)(q15;q24)[9]

0635-1	48	N/A	46, XX, der (10), del(12)(q13 or q14) [18]

0643-1	52	1.0	n.d.
0643-2	52	6.0	46, XX, t(12;14)(q15;q24)[14]
0643-3	52	2.0	46, XX [12]

0646-1	47	9.5	46, XX, t(2;12)(p21;p13)[11]

0649-1	42	2.0	46, XX [14] remark: 46, XX, der(14)t(12;14) as single cell aberration

0653-1	50	1.0	46, XX [14]
0653-2	50	1.5	46, XX [15]
0653-3	50	2.5	46, XX [10]

0654-1	43	3.0	46, XX [8]

0654-2	43	2.3	47, XX, +12 [4]/46, XX [12]

0654-3	43	1.8	46, XX [14]

0668-1	57	3.0	46, XX [10]
0668-2	57	2.0	46, XX [11]
0668-3	57	2.5	46, XX [7]

0673-2	45	3.0	46, XX [13]

0681-1	48	7.5	45, XX, der(1)( ?)t(1;14)(p36.3;q24), der(1)del(1)(q32)?t(1;11)(p36.1;q13), del(3)(q26), add(6)(p21.3), -10, -11, del(12)(q24.1), der(14)t(6;14)(p21.3;q24), add(19)(q13.4), +r [20]

0682-1	69	1.5	46, XX [16]
0682-2	69	1.0	46, XX [38]

0683-1	47	6.0	46, XX, del(7)(q22q32)[5]/46, XX [3]
0683-2	47	1.0	46, XX [21]

0686-1	57	6.0	n.d.
0686-2	57	1.0	46, XX [20]
0686-3	57	1.0	46, XX [13]
0687-1	N/A	1.0	46, XX, der(10)add(10)(p)add(10)(q)[3]/46, XX [15]

0694-2	N/A	9.0	n.d.

0695-1 0695-2 0695-3	68 68 68	2.5 6.0 1.0	n.d. n.d. n.d.

0700-1	N/A	1.5	46, XX [11]

Karyotypes are described according to [20].

### Treatment by Nutlin-3

For treatment by nutlin-3 (Biomol, Hamburg, Germany) tissue samples were minced into small pieces of approximately 0.5 cm diameter and incubated in medium 199 supplemented with 20% FCS and nutlin-3 (3, 10, or 50 μM) for 72 h. As controls tissue explants were incubated in medium 199 supplemented with 20% FCS without nutlin-3 for 72 h.

### RNA Isolation

RNA isolation was performed using the RNeasy mini kit (Qiagen, Hilden, Germany) and DNase I digestion was performed following the manufacturer's instruction.

### cDNA-Synthesis

About 250 ng of total RNA were reverse transcribed with 200 U/μl of M-MLV reverse transcriptase (Invitrogen, Karlsruhe, Germany), RNase Out, 150 ng random hexamers and 10 mM dNTPs according to the manufacturer's instructions. RNA was denatured at 65°C for 5 min and subsequently kept on ice for 1 min. After adding the enzyme to the RNA primer mixes, samples were incubated for 10 min at 25°C to allow annealing of the random hexamers. Reverse transcription was performed at 37°C for 50 min followed by inactivation of the reverse transcriptase at 70°C for 15 min.

### Quantitative Real-Time PCR (qRT-PCR)

Relative quantification of transcription levels was carried out by real-time PCR analyses using the Applied Biosystems 7300 real-time PCR system (Applied Biosystems, Darmstadt, Germany). Commercially available gene expression assays (Applied Biosystems, Darmstadt, Germany) were used for quantification of mRNA of human *p14^Arf ^*(Hs00924091), *BAX *(Hs00180269_m1), *CDKN1A *(Hs99999142), *MDM2 *(Hs01066930_m1) and *MKI67 *(Hs00606991_m1). *HPRT *served as endogenous control [7]. All qRT-PCR experiments were done in triplicate.

### Cytogenetic and Molecular Cytogenetic Studies

Chromosome analyses and fluorescence in situ hybridization (FISH) were performed following routine techniques as described previously [11].

### Immunohistochemical studies of tissue explants

Immunohistochemical staining for p53 (clone DO-7, DAKO, Glostrup, Denmark) as well as Ki-67 (clone MIB-1, DAKO, Glostrup, Denmark) proteins was performed using a detection kit (DAKO ChemMate, DAKO, Glostrup, Denmark) and a semi-automated stainer (DAKO TechMate, DAKO, Glostrup, Denmark) according to the specifications of the manufacturer. For antigen retrieval the slides were treated in a PT Link module (DAKO, Glostrup, Denmark) using the EnVision™ FLEX Target Retrieval Solution, Low pH (DAKO, Glostrup, Denmark). For both antibodies, the dilution was 1:100.

### Western Blot Analysis

Protein extracts were obtained using RIPA buffer and concentrations were determined using a BCA protein assay (Thermo Scientific, Rockford, USA) according to the manufacturer's instructions. Total protein (16 μg per lane) was separated using a SDS-polyacrylamide gel and transferred onto a nitrocellulose membrane. SeeBlue Plus2 Pre-Stained Standard (Invitrogen, Karlsruhe, Germany) was used as marker. The membranes were incubated with primary anti-p53 (mouse, monoclonal, 1:200; DAKO, Hamburg, Germany) and anti-beta-actin (rabbit, polyclonal, 1:7, 500; Santa Cruz Biotechnology, California, USA) followed by incubation with the corresponding secondary antibodies (alkaline phosphatase-conjugated goat anti-mouse IgG (1:5, 000) (Invitrogen, Karlsruhe, Germany) and alkaline phosphatase-conjugated bovine anti-rabbit IgG (1:3, 750) (Santa Cruz Biotechnology, California, USA)). The bands were visualized by incubating the membrane with NBT/BCIP (Nitro blue tetrazolium chloride/5-Bromo-4-chloro-3-indolyl phosphate) (Roche Applied Science, Mannheim, Germany) according to the manufacturer's instructions. Immunoblots were scanned using a flatbed scanner and image analysis was performed with the ImageJ gel analysis algorithm.

### Statistical Analysis

The statistical significance of differences was assessed by the student's *t *test. In all comparisons, p < 0.05 was considered being statistically significant and p < 0.01 was considered being highly significant. Individual differences between the expressions in leiomyomas and myometrium were tested by a pairwise t test, using means of measurements within an individual if more than one leiomyoma was analysed for that individual.

## Results

### Nutlin-3 induces senescence as well as apoptosis in tissue explants from leiomyomas

**Figure 1:**
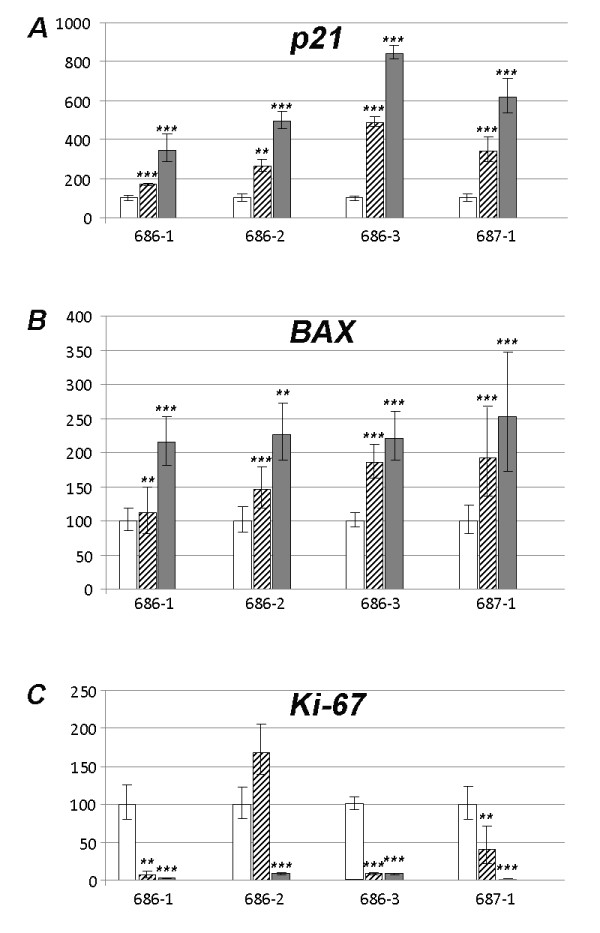
**Tissue explants taken from uterine fibroids show a nutlin-3 sensitivity as revealed by the increased expression of p21, BAX, and decreased expression of Ki-67 mRNA after 72 h incubation with nutlin-3 compared to the controls**. Expression in the control explants (white bars, no treatment) was always set 100%. Ordinate: % change of expression compared to control. Hatched bars: 3 μM nutlin-3; grey bars: 10 μM nutlin-3. For tumor numbers below each group of bars rf. to Table 1. A: p21, B: BAX, C: Ki-67. Statistically significant increases (*p21 and BAX*) or decreases (*Ki-67*) are given by asterisks ( **, p < 0.01; ***, p < 0.001).

To see if nutlin-3 can induce similar effects on the gene expression of *p21 *and *BAX *as were recently observed explants from a total of four leiomyomas (Table 1) were incubated with nutlin-3 (3 μM and 10 μM each) for 72 h and then checked for the expression of these genes. In addition, the expression of the proliferation marker *Ki-67 *was analyzed. Compared to the controls the results show concentration-dependent highly significant (p < 0.01 or p < 0.001, respectively) increases of the expression of *p21 *and *BAX *in all explants analyzed (Figure 1A, B) with both concentrations used. Accordingly, the expression of *Ki-67 *strongly decreased except for one case where with a concentration of 3 μM an increase of *Ki-67 *expression was noted (Figure 1C).

### Nutlin-3 increases the amount of p53 in a concentration-dependent manner

**Figure 2:**
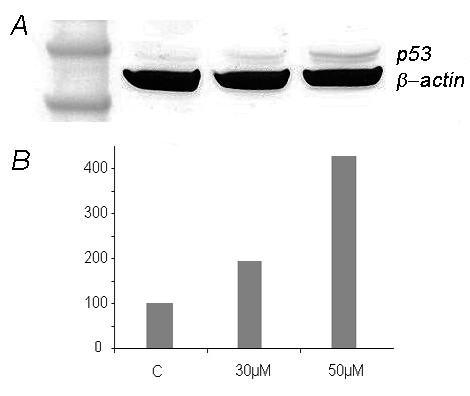
**Western Blot analyses of explants treated with nutlin-3 reveal a concentration dependent increase of the amount of p53**. A: Western Blot analysis of p53 of explants from an UL (case 700-1) treated with 30 μM and 50 μM nutlin-3 for 72 h shows a concentration-dependent increase of the amount of p53. Lane 1: marker SeeBlue Plus2 Pre-Stained Standard (Invitrogen, Karlsruhe, Germany), lane 2: control without nultin-3, lane 3: 30 μM nutlin-3, lane 4: 50 μM nutlin-3 (left to right). B: p53 protein expression determined after immunoblotting (c.f. A) by ImageJ (as described in the materials and methods section) against beta-actin. Control was set 100%. Ordinate: % change of p53 expression compared to control.

Nutlin-3 acts as an inhibitor of MDM2 which in turn destabilizes p53. To see if the increased expression of the senescence and apoptotic markers that was noted after treatment of the explants with nutlin-3 was indeed due to an elevated level of p53 explants of case 0700-1 treated with 30 μM and 50 μM nutlin-3, respectively, for 72 h were used for immunoblot analyses. The results clearly show a concentration-dependent increase of p53 (Figure 2A, B).

### Fibroids express significantly higher levels of *p14^Arf ^*than matching myometrium

**Figure 3:**
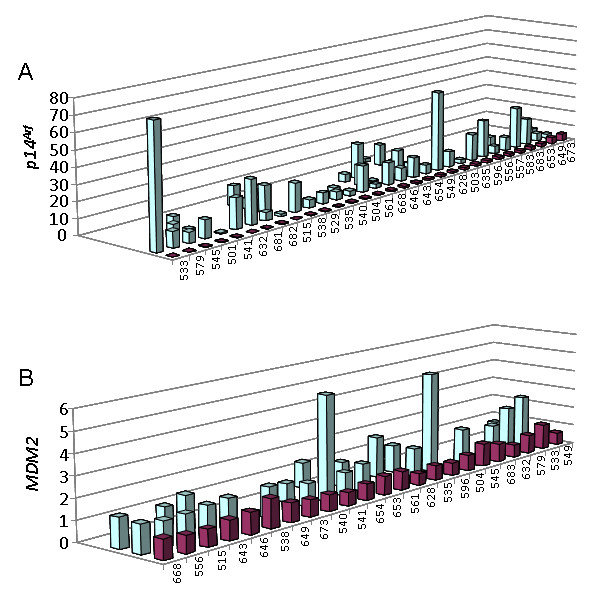
**Leiomyomas usually express higher levels of *p14^Arf ^*and *MDM2 *than matching myometrium**. Columns within each row give the relative expression of p14^Arf ^mRNA (A) and MDM2 mRNA (B) in myometrium (violett columns) and matching fibroids (blue columns) from one patient each as revealed by qRT-PCR. Number below each row corresponds to the patient's lab no. The corresponding leiomyomas are depicted in a numerical order (cf. Table 1). Ordinates gives relative expression of p14^Arf ^and MDM2, respectively.

**Figure 4:**
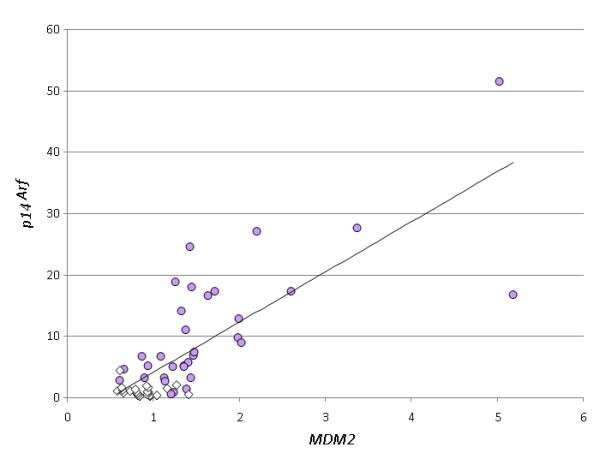
**Highly significant (p < 0.001) correlation between the *MDM2 *expression (x-axis) and the *p19^Arf ^*expression (y-axis) in myometrium (white boxes) and leiomyoma tissues (violet circles)**. One myometrial tissue served as calibrator (expression: 1).

Previously, expression analysis of a series of UL and eight myometrial samples revealed a significantly higher expression of p14^Arf ^in the UL compared to their tissue of origin [7]. Now, we have extended that smaller series to a total of 52 fibroids and 31 matching myometrial tissues. On average, the myometrial samples expressed 10-fold lower levels of p14^Arf ^than the fibroids (1.14 vs. 11.5) (p < 0.001). Moreover, in all but two fibroids the expression of p14^Arf ^in the fibroid taken from a patient extended that of the corresponding myometrium (Figure 3A). Also, a marked heterogeneity of the expression between the UL of individual patients was noted as well. Because we were recently able to demonstrate that the expression of p14^Arf ^is correlated with that of MDM2 [7] most likely resulting from a positive feedback-loop driven by p53 we have also compared the expression of MDM2 in fibroids and matching myometrium. In all samples examined the expression of MDM2 in the fibroids exceeded that found in the myometrium (Figure 3B). Furthermore, we were able to confirm the positive correlation between the expression of *p14^Arf ^*and *MDM2 *(Figure 4) that was noted before on a smaller series of cases including three myometrial tissues only [7].

### As a rule leiomyomas show a higher sensitivity against nutlin-3 treatment than matching myometrium

**Figure 5:**
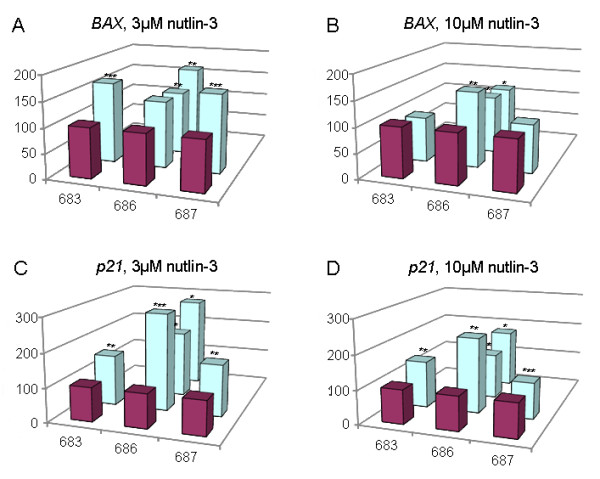
**As a rule fibroids display a higher nutlin-3 sensitivity than matching myometrium**. Explants from five fibroids from three patients were checked for their nutlin-3 sensitivity after incubation with 3 μM or 10 μM nutlin-3 respectively, for 72 hours. As an indicator for sensitivity the expression of BAX (A, B) and p21 (C, D) mRNA was determined by qRT-PCR. Myometrium (violet columns) was always set 100% and the expression of the corresponding fibroids (light blue columns) refers to that value. Numbers below each row indicate the patient's lab no. (cf. Table 1). The corresponding leiomyomas are depicted in numerical order. Statistically significances are given by asterisks (*, p < 0.05; **, p < 0.01; ***, p < 0.001).

In an earlier paper we had been able to show that leiomyomas show elevated levels of p14^Arf ^mRNA compared to myometrium likely to result in an elevated level of p53 which due to a negative feedback-loop between p53 and MDM2 in turn leads to an activation of MDM2 [7]. Furthermore, we had hypothesized that within the p14^Arf^-driven pathway a delicate balance between p53 and MDM2 assures the proliferative activity of leiomyomas as well as their genomic integrity [12]. Accordingly, it can be speculated that leiomyomas may be more sensitive against MDM2 inhibition than matching myometrium. For the same cases used before and one additional case (one leiomyoma plus matching myometrium; cf. Table 1) samples of myometrial tissue have been treated with 3 μM and 10 μM nutlin-3 for 72 h to analyze and compare the expression of *p21, and BAX *with that of the matching leiomyomas. The expression of *p21 *in the leiomyomas generally exceeded that found in the myometrium with 3 μM nutlin-3 (211.93% vs. 100%; p < 0.01) and 10 μM nutlin-3 (150.84% vs. 100%; p < 0.05). Moreover, in all three cases and with two concentrations the expression of *p21 *in the explants from the single fibroids exceeded that determined in the matching myometrium Figure 5A, B). Likewise, the expression of *BAX *in the tumors exceeded that seen in the myometrium (3 μM nutlin-3: 145.37% vs. 100%; p < 0.001; 10 μM nutlin-3: 112.85% vs. 100%; n.s.). As to the BAX expression of the single tumors a higher sensitivity of the leiomyomas compared to their matching myometrium was noted except for two cases (683 and 687) where with 10 μM the expression in the myometrium slightly exceeded that of the leiomyoma (Figure 5C, D). Except for the *BAX *expression of these latter two explants and one other explant all individual differences between any of the other explants and their matching myometrium samples were found to be statistically significant. In addition individual differences between the expressions in leiomyomas and myometrium were tested by a pairwise t test, using means of measurements within an individual if more than one leiomyoma was analyzed for that individual. This test procedure resulted in a highly significant difference (p < 0.01) for the expression of BAX and a borderline significance (p = 0.06866) for the expression of *p21*.

### Nutlin-3 treatment induces an increasing amount of p53

**Figure 6:**
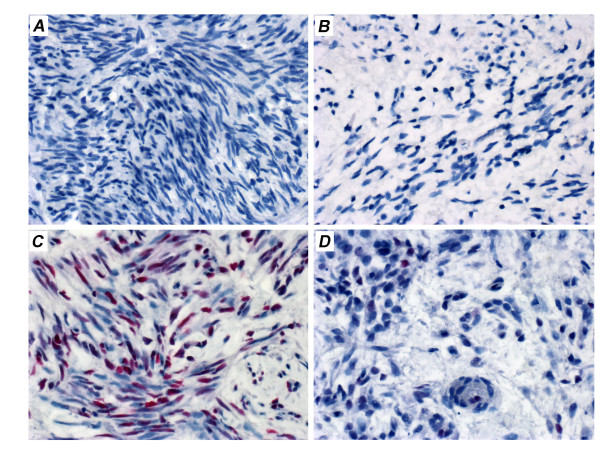
**Immunohistochemistry reveals that nutlin-3 causes a dose-dependent increase of p53 in fibroids exceeding that in myometrium**. A: leiomyoma 695-3, control without nutlin-3; B. myometrium 695-0, control without nutlin-3; C: leiomyoma 695-3 after 6 days exposure to 10 μM nutlin-3; C: myometrium 695-0 after 6 days exposure to 10 μM nutlin-3.

Because MDM2 inhibition can be expected to raise the level of p53 and it was shown that nutlin-3 treatment increases the amount of p53 expressed by the explants (c.f. Figure 2) we were interested to see if an IHC-scoring system may also detect the comparable differences between UL and matching myometrium as those shown by qRT-PCR for the expression *p21 *and *BAX*. Treatment of the explants by nutlin-3 caused a clearly increased intensity of p53 staining which was concentration-dependent (Table 2). An example of the p53 staining patterns after IHC is given in Figure 6. Further analyses showed an increased number of p53-positive cells as well when comparing UL with matching myometrium. In some of the samples IHC using Ki-67 was performed as well. Whereas, corresponding to the literature [13], 1 - 2% positive cells were seen in the controls, they were absent from any of the nutlin-3-treated samples.

**Table 2:** Treatment by nutlin-3 results in a concentration dependent increase of the number of p53 positive cells as well as of the staining intensity as revealed by immunohistochemistry.

#case	treatment	duration of treatment	number of p53-positive cells	intensity	percentage of Ki67-positive cells
**0694-2**	control		11	0 - 1	n.d.
			
	10 μM nutlin-3	72 h	393	2	n.d.
			
	30 μM nutlin-3		1, 277	3	n.d.

**0695-0**	control		0	0	4%
			
	10 μM nutlin-3		11	1	< 1%
			
	30 μM nutlin-3		188	1	< 1%

**0695-1**	control		0	0	-
			
	10 μM nutlin-3		42	0 - 1	-
			
	30 μM nutlin-3	6 days	89	1	-

**0695-2**	control		0	0	< 1%
			
	10 μM nutlin-3		194	1 - 2	-
			
	30 μM nutlin-3		799	3	-

**0695-3**	control		0	0	< 1%
			
	10 μM nutlin-3		770	3	
			
	30 μM nutlin-3		1, 493	3	

**0687-0**	control		0	0	n.d.
			
	10 μM nutlin-3		2	1	n.d.
			
	30 μM nutlin-3	72 h	257	2	n.d.

**0687-1**	control		0	0	n.d.
			
	10 μM nutlin-3		398	2	n.d.
			
	30 μM nutlin-3		n.d.	n.d.	n.d.

For further information of the tumors studied (karyotype, age, tumor size) see Table 1.

### Uterine leiomyoma cells do not recover during long-term inhibition of MDM2

**Figure 7:**
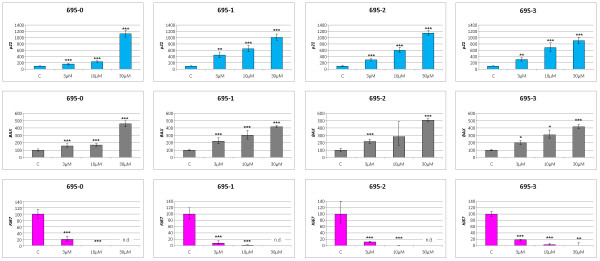
**After incubation with nutlin-3 for six days, an increased expression of *p21 *as well as *BAX *and a decreased expression of *Ki-67 *compared to the controls was noted**. Myometrium (C, not treated) was always set 100% and the expression of the corresponding fibroids refers to that value. A: expression of p21 mRNA, B: expression of BAX mRNA, C: expression of Ki-67 mRNA. For sample numbers refer to Table 1. Statistically significant increases (*p21*, and *BAX*) or decreases (*Ki-67*) are given by asterisks (*, p < 0.05; **, p < 0.01; ***, p < 0.001). n.d.: not detectable.

To see how the gene expression patterns for *p21, BAX*, and *Ki-67 *change after six days of MDM2 inhibition and if the cells become resistant we have additionally performed gene expression analyses for these genes on a series of three UL along with matching myometrium (Figure 7A-C). Akin to a shorter exposure to nutlin-3 the long term experiment did also significantly increase the expression of *p21 *and *BAX *and decreased that of *Ki-67 *in a dose-dependent manner. Furthermore, clear differences between the myometrium and any of the matching fibroids became apparent that again point to a reduced sensitivity of the myometrium against the MDM2 inhibition.

## Discussion

Recently, we have shown that compared to myometrium leiomyomas express higher levels of p14^Arf^, an important upstream regulator of p53 [7]. Herein, this observation has been confirmed on a much larger series of cases. In addition, a marked heterogeneity of the p14^Arf ^expression was noted when comparing UL from one patient. Most likely due to the well-documented positive feedback-loop that exists between p53 and MDM2 [14,15], the fibroids do also express higher levels of MDM2 leading to a positive correlation between *p14^Arf ^*and *MDM2 *expression [7] that may keep fibroids in a delicate balance between growth and senescence. This finding has been confirmed here based on a larger series and makes MDM2 an interesting target molecule for the growth control of leiomyomas. Accordingly, we were able to demonstrate that in leiomyoma cells *in vitro *treatment with nutlin-3, a small-molecule inhibitor of MDM2, activates the expression of canonical groups of genes associated with senescence and apoptosis downstream of p53 [9]. Herein, we were able to show that as a rule tissue explants taken from leiomyomas display a higher sensitivity after treatment with nutlin-3 than do those from matching myometrium. Of note, nutlin-3 increased the expression of p53-dependent marker genes associated with senescence as well as with apoptosis. As a rule for both genes i.e. *p21 *and *BAX *leiomyoma tissue turned out to be more sensitive than myometrial tissue. We assume that the higher expression of *p14^Arf ^*in the fibroids [7] is the most likely explanation for this different sensitivity. When interpreting leiomyomas as the result of proliferation of a stem-cell like population it seems reasonable to assume that this proliferation is accompanied by activation of the p53-pathway *via *p14^Arf ^to protect the cellular genome [12]. *Vice versa*, functional p53 activates the positive feedback-loop with MDM2 that may be essential for the development of fibroids. Likewise, disturbing this balance by MDM2 inhibitors can be assumed to cause senescence as well as apoptosis of the leiomyoma cell population because p53 still remains intact with a strongly decreased MDM2 activity.

The present study has limitations which are mainly related to the use of an *in vitro *model. Cell cultures from fibroids can easily be set up without major problems of overgrowing of normal cells but the cells have only a limited growth potential and, as an even more serious reason for concern, rapidly lose characteristic features of leiomyomas *in vivo*. E.g. the estrogen receptor level of fibroid cells rapidly declines *in vitro *[16]. Immortalization of the cells is possible e.g. by using SV40-large T-antigen or the SV40 early region, but with these cells experiments are still facing the problem of drastic changes compared to the normal cells [17]. Though the explants cultures as used here may reflect the *in vivo *situation better than isolated cells it is well known that explant cultures as well loose characteristics of the tissue in vivo e.g. a decrease the expression of estrogen receptors after a short time *in vitro *has been noted [18] that was within the time-range used here for the nutlin-3 experiments. Nevertheless, the higher sensitivity of the leiomyoma tissue against the inhibition of MDM2 compared to surrounding myometrium corresponds to a higher in vivo expression of p14^Arf ^and thus likely exists in vivo as well. Then, it may have considerable therapeutical implications. In UL antagonizing MDM2 seems to be a way to induce growth arrest as well as apoptosis. Both can be expected to irreversibly impair tumor growth and to decrease the tumor size, respectively. Interestingly, estrogens are known as negative regulators of p53 [19]. Thus, it seems reasonable to speculate that changes of the behaviour of fibroids following changes of the hormonal milieu as in particular their shrinkage are at least in part also due to skewing the balance towards p53. Accordingly, a combination e.g. of a GnRH antagonist and a MDM2 antagonist may be a favourable approach for the treatment of fibroids. In summary, the results of the present study strengthen the idea that senescence and apoptosis play an important role in the growth control of fibroids and that their induction may offer interesting approaches for the therapy of these frequent tumors.

## Conclusions

Based on their expression of p14^Arf ^we have concluded that as a rule leiomyomas represent a cell population of advanced senescence compared to matching myometrial tissue. Accordingly, fostering the p14^Arf ^- p53 network by nutlin-3, a known MDM2 antagonist, increases the expression of pro-senescence and pro-apoptotic genes in tissue explants from myometrium as well as from leiomyomas with the latter displaying a significantly higher sensitivity than the matching normal tissue. In summary, though the data need confirmation in vivo they point to members of the p53-network as to potential novel therapeutic targets for the treatment of uterine fibroids.

## Competing interests

The University of Bremen is currently applying for a patent claiming the use of MDM2 inhibitors for the treatment of uterine fibroids.

## Authors' contributions

DNM contributed to the conception and design of the study, the acquisition of data, the analysis and interpretation of data and to the manuscript writing. BMH contributed to the conception and design of the study, the analysis and interpretation of data and revised the manuscript critically for important intellectual content. AR and JF participated in data acquisition. GB and SB contributed to the analysis and interpretation of data. WW did the statistical analyses. ICJ and UD contributed to the provision of study material. JB contributed to the conception and design of the study, the analysis and interpretation of data, the manuscript writing and revised the manuscript critically for important intellectual content. All authors read and approved the final manuscript.

## Pre-publication history

The pre-publication history for this paper can be accessed here:

http://www.biomedcentral.com/1472-6874/12/2/prepub
